# Over-the-counter drug consumption and related factors, evidence from the European Health Interview Survey

**DOI:** 10.1080/20523211.2025.2455068

**Published:** 2025-02-03

**Authors:** Aida Isabel Tavares

**Affiliations:** a CEISUC – Centre for Health Studies and Research, University of Coimbra, Coimbra, Portugal; b CiBB – Center for Innovative Biomedicine and Biotechnology, University of Coimbra, Coimbra, Portugal; c ISEG, UL – Lisbon School of Economics and Management, University of Lisbon, Lisbon, Portugal

**Keywords:** OTC drugs, consumption, drivers, market deregulation, Europe

## Abstract

**Background:**

The aim of this work is to find the factors related to over-the-counter drug (OTC) consumption in Europe considering the differences in OTC market regulation.

**Methods:**

A sample obtained from the European Health Interview Survey (EHIS) of 2019 is used to estimate a logistic regression. This sample includes 286,413 people from 26 countries. The outcome variable is derived from the question about the use of non-prescribed drugs. The independent variables include a set of predisposing, enabling, and need factors. Finally, countries with similar OTC retail market regulations are considered to control for heterogeneity in this regard.

**Results:**

The main findings show that OTC market deregulation is not strictly related to the share of OTC consumption. Despite some heterogeneity based on the type of OTC retail market regulation, there is a set of common drivers for its use by people across countries, such as age, gender, education, suffering from chronic disease or pain and being on a waiting list for medical treatment. When considering individual country and cluster of countries controls, there are some relevant results such as the role played by accessibility to pharmacies and OTC retailers; the positive correlation between OTC drugs and prescribed drug consumption, and the positive correlation between unmet health care needs due to financial household constraints and OTC drugs use.

**Conclusions:**

There is no clear relationship between OTC drug use and OTC retail market regulation. There are several predisposing, enabling, and need factors that promote the use of OTC drugs. The relevant policies relate to the inequal access to OTC drugs across countries that may result from different market regulations and different consumption drivers.

## Background

Over-the-counter (OTC) drugs are medicinal products chosen and used by consumers to treat self-diagnosed illnesses without the direct support of a health professional (Rutter, 2015; WHO, 2000, 2023). The use – and consumption – of OTC drugs provide some advantages for different stakeholders: consumers save time and money as they have easier access to medical treatment while also feeling empowered to look after their own health; doctors save time by not having to treat minor ailments or ailments like pain, colds or the flu, and allergies; and society benefits from lower rates of work absenteeism and higher productivity. Despite these benefits, there are some serious concerns related to the consumption of OTCs. These disadvantages include polypharmacy, adverse effects and unwanted hospital admissions, incorrect diagnosis and inappropriate dosage, drug dependency and deliberate misuse, and delayed diagnosis for a severe health condition (AESGP Foundation, 2004; Ghosh et al., 2015; Hughes et al., 2001; Noone & Blanchette, 2018; WHO, 1998).

There was a clear trend towards the promotion of self-medication around the end of the 1990s to the early 2000s (WHO, 2000), a factor which contributed to the increase in demand for OTC medicines. This upwards trend in demand was paralleled by another on the supply side characterised by the ‘Rx-to-OTC switch’, i.e. a transfer of well-established prescription medications (Rx) to over-the-counter (OTC) drugs (Cohen et al., 2013; Rosenau, 1994).

Accompanying these trends of self-medication and the Rx-to-OTC switch was another related to market liberalisation (Mahecha, 2006; Oleszkiewicz et al., 2021; Vila et al., 2023; Vogler et al., 2014) in Europe and in the USA. This market liberalisation refers mainly to wholesale and retail parts of the pharmaceutical market. Market liberalisation in Europe accounts for several dimensions of the overall regulatory framework such as pricing, pharmacy ownership, and OTC drug selling channels; generic drug entry and incentives to use them; and also consumer access regulations which include number of pharmacies per capita, opening hours, online services and other services provided by pharmacies (Oleszkiewicz et al., 2021; Vila et al., 2023; Vogler et al., 2014). Consequently, there is considerable heterogeneity in market regulations (Oleszkiewicz et al., 2021; Vila et al., 2023) across European countries which may influence the supply and availability of OTC drugs.

It is possible to group European countries according to their legal framework for selling these drugs. Using a regulation framework based on four parameters – price, ownership, distribution, and dispensing restrictions – Vila et al. (2023) revised the regulation of 30 European countries and classified the countries into 2 broad groups: (i) countries where the dispensing restriction imposes retailing exclusively through pharmacies (e.g. Germany, Finland, and Belgium), called ‘Pharmacy retail only’, and (ii) countries where drug retail can take place in pharmacy as well as non-pharmacy sites (e.g. the Netherlands, Sweden, and Italy), called ‘Pharmacy and Non-pharmacy retail’. Additionally, it is possible to identify clusters of countries which benefit from an identical market regulation regime, based on the same four parameters mentioned above. Seven clusters of countries are thus identified: Clusters 1–3 are in the group ‘Pharmacy retail only’, identified mainly according to the price restrictions and distribution modes, while Clusters 4–7 are in the group ‘Pharmacy and Non-pharmacy retail’, mainly defined by similar dispensing rules, ownership, and distribution Vila et al. (2023). The countries in each cluster are listed in Table 1.

**Table 1.** Independent variables.

Variables name and label	Description
**Predisposing factors**	
male	Equals 1 if respondent is male; 0 if female
age	Equals the median value of the interval of age groups
education	Equals the number of years of completed education
family_size	Equals the number of people in the household
civil status	
single	Equals 1 if respondent is single; 0 otherwise
married	Equals 1 if respondent is married; 0 otherwise
divorced	Equals 1 if respondent is divorced; 0 otherwise
widow	Reference category
area of residence	
city	Equals 1 if respondent resides in cities; 0 otherwise
rural	Equals 1 if respondent resides in rural areas; 0 otherwise
towns	Reference category (includes suburbs)
**Enabling factors**	
income quantil	
Q1 (poorest)	Equals 1 if respondent income is within first quantile; 0 otherwise
Q2	Equals 1 if respondent income is within second quantile; 0 otherwise
Q3	Equals 1 if respondent income is within third quantile; 0 otherwise
Q4	Equals 1 if respondent income is within fourth quantile; 0 otherwise
Q5 (richest)	Reference category
labour status	
employed	Equals 1 if respondent is employed; 0 otherwise
unemployed	Equals 1 if respondent is unemployed; 0 otherwise
retired	Equals 1 if respondent is retired; 0 otherwise
student	Equals 1 if respondent is student; 0 otherwise
other	Reference category
rx_drugs	Equals 1 if respondent took prescribed drugs during the last two weeks (excluding contraception); 0 otherwise
accessibility/proximity	
walk_nrdays	Equals the number of days in a typical week the respondent walks to get to and from places at least 10 min continuously; 0 otherwise
bike_nrdays	Equals the number of days in a typical week the respondent bicycles to get to and from places at least 10 min continuously; 0 otherwise
**Needs**	
self-assessed health	
SAH_VGood	Reference category
SAH_Good	Equals 1 if respondent assesses health status as good; 0 otherwise
SAH_Fair	Equals 1 if respondent assesses health status as fair; 0 otherwise
SAH_Bad	Equals 1 if respondent assesses health status as bad; 0 otherwise
SAH_VeryBad	Equals 1 if respondent assesses health status as very bad; 0 otherwise
chronic_disease	Equals 1 if respondent reports suffering from at least one chronic illness or a long-standing health problem; 0 otherwise
pain	Equals the intensity of body pain the respondent felt during the last four weeks: from 0 – no pain to 6 – very severe pain
unmet needs	
unmet_waiting	Equals 1 if respondent reports unmet need for health care in the last 12 months due to long waiting list; 0 otherwise
unmet_distance	Equals 1 if respondent reports unmet need for health care in the past 12 months due to distance or transportation problems; 0 otherwise
unmet_financial	Equals 1 if respondent reports incapacity to afford medical examination or treatment in the past 12 months; 0 otherwise
bmi	Equals the body mass index of respondent
**Countries**	**Country codes are displayed in Table A2, in Appendix**
Regulatory framework	
pharmacy retail only	
Cluster1	Austria, Cyprus, Greece, Estonia, Germany, and Spain
Cluster2	Finland and Luxembourg
Cluster3	Belgium, Bulgaria, Latvia, Malta, and Slovakia
pharmacy and non-pharmacy retail	
Cluster4	Denmark, Hungary, Lithuania, and Sweden
Cluster5&6	Croatia, Italy, Slovenia, Romania, and Portugal
Cluster7	Czechia, Ireland, Netherlands, and Poland

In countries with ‘Pharmacy retail only’, OTC drugs can only be obtained in community pharmacies, and competition, if any, occurs as established by regulation. On the other hand, in countries with ‘Pharmacy and Non-pharmacy retail’ there was a shift towards a market liberalisation that affects several features of the market, such as prices, quantities and varieties available, quality of service, variety of services offered by retailers, access and delivery of drugs. Therefore, market liberalisation has an impact on consumer welfare, as well as the overall well-being and benefits that individuals derive from the consumption of drugs and pharmacy services (Cohen et al., 2013; Oleszkiewicz et al., 2021; Vogler et al., 2014).

**Figure 1.**
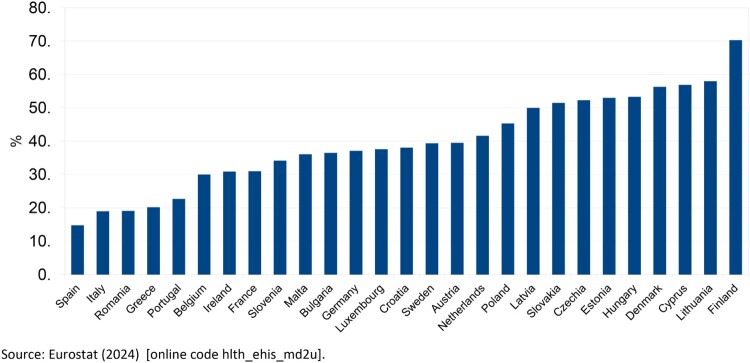
Self-reported use of OTC medicines in Europe, 2019. Source: Eurostat (2024) [online code hlth_ehis_md2u].

**Figure 2.**
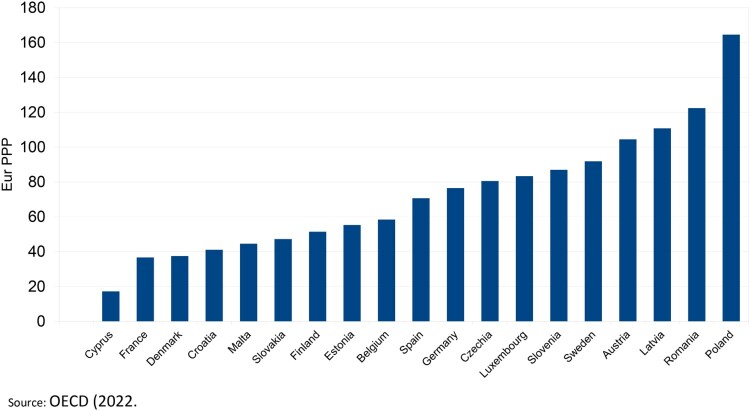
Expenditure on retail OTC medicines per capita, 2020 (or nearest). Source: OECD (2022).

Consumption of OTC in Europe is varied, whether in the use of these kinds of drugs or in the expenditure per capita, as shown in Figures 1 and 2. While Southern European countries – Spain, Italy, Greece, and Portugal – self-report low percentages of use of OTC drugs, Northern countries, such as Finland, Lithuania, and Denmark, are more inclined to use them. Nevertheless, there is no clearly identifiable geographic trend concerning the use of OTC drugs across countries (Figure 1).

Despite the incomplete information regarding expenditure per capita in OTC drugs, the heterogeneity is still observable and is not correlated with the use of these drugs as described above. While countries such as Cyprus, France, and Denmark display a low level of expenditure, the opposite can be seen in Latvia, Romania, and Poland (Figure 2).

Studies on the consumer benefit derived from the liberalisation of the retail pharmaceutical market are scarce and conclusions on the effect on accessibility, prices, quantities consumed, and consumer welfare have been mixed. Concerning accessibility and number of pharmacies, liberalisation may lead to new players entering the market (Moura & Barros, 2020), but usually, entry is concentrated in urban areas (Vogler et al., 2014), or it may evolve to concentration forms which limit competition and entry (Anell 2005; Pisek, 2017). Concerning the benefit to the consumer, two studies, one in South Korea and the other in Portugal, show that the deregulation of the OTC drugs market resulted in lower average prices (Moura & Barros, 2020; Wooyong et al., 2022). One may therefore expect an increase in demand for these drugs according to the law of demand in economics, depending on price elasticity and cross-price elasticity. Evidence from nine European countries (Vogler et al., 2014) and from Germany (Stargardt et al., 2007), on the other hand, indicates that after market deregulation, the decreasing effect on prices was very small or non-existent, leading one to expect a negligible effect on consumption. Summing up, little is known about the dynamics of OTC pharmaceutical retail markets after liberalisation.

There are some empirical studies that aim to understand the drivers for the use of OTC drugs and self-medication in Europe, the USA, and in low-medium income countries. The predictors for self-medication were well-described and explained in a systematic review published in 2014 (Shaghaghi et al., 2014). A recent study carried out in the USA (Rashrash et al., 2021) used the Andersen model for Health Services Use to identify the factors associated with pharmacy selection by consumers. In Europe, several studies have been carried out for Austria (Mayer & Österle, 2015), Spain (Daban et al., 2010; Jiménez-Rubio & Hernández-Quevedo, 2010), Portugal (Tavares, 2013; Tavares et al., 2022), Greece (Kamekis et al., 2020), Serbia (Tripković et al., 2018), Romania (Tarciuc et al., 2020) and the UK (Green et al., 2016; Porteous et al., 2015), and there is also research for Central Eastern European countries (Vogler et al., 2015). These studies may use the Andersen conceptual model or be based on an *ad-hoc* approach. Among these studies, worth noting is the Portuguese study (Tavares et al., 2022) that uses the National Health Survey – which is the national application of the EHIS (European Health Interview Survey) – and uses an empirical model based on the Andersen framework. Finally, also worth mentioning is the work undertaken by Vogler et al. (2014), who have used average distance, which differs from country to country, as an indicator of pharmacy accessibility. In this way, accessibility may be taken as an enabling factor for medicine consumption for which it is hard to collect empirical evidence.

The aim of this study is to identify the drivers of the consumption of OTC drugs in several European countries and to explore possible different profiles of drivers in different retail market regulations. Little work has been done on this topic at the European level and none accounted for the regulatory characteristics of the OTC drugs retail market. We use European Health Interview Survey (EHIS) data for 26 European countries, estimate a logistic regression for OTC consumption, and explore the differences in regulation. The contribution of this study is unique: by combining the consumer perspective and supply regulation of OTC drugs, it provides evidence on possible inequalities in OTC drugs access across European countries.

## Methods

### Conceptual model

The conceptual model supporting our analysis is Andersen’s model for Health Services Use (Andersen, 1968, 1981). The model has been used in several studies, including those associated with self-care. The model considers three major components that interact among themselves and which influence the use of health services at the individual and contextual levels. Those three components define (i) predisposing, (ii) enabling, and (iii) need factors which influence the decision of people to buy from pharmacies and other retailers. Predisposing factors mainly include demographic and community characteristics; enabling factors are related to financing and organisational characteristics which enable and facilitate the use of pharmacies and OTC retailers; needs are factors that result in the action to self-medicate and to consume OTC medicines, which are related to the type and duration of illness or symptoms.

### Data and sample

Data was collected from the EHIS wave 3 from 2019. The sample comprises 286,413 people from 26 countries. The legal framework for the EHIS is defined in Regulation (EC) No 1338/2008 of the European Parliament and of the Council of 16 December 2008 on Community statistics on public health and health and safety at work. Samples for each country are based on a nationally representative probability sample of people aged 15 and over, residing in private households in the member states. The model questionnaire is designed for a face-to-face interview mode (Interviewer-PAPI and CAPI) (Eurostat, 2020). The EHIS data are available from Eurostat upon request and access to the data used in this study was granted under research project (RPP 322/2022-EHIS).

### Outcome variable

The outcome variable (named OTC) is obtained from the survey question ‘During the past two weeks, have you used any medicines or herbal medicines or vitamins not prescribed by a doctor?’. The OTC variable takes a value 1 if the answer was ‘yes’ and ‘0’ otherwise. This variable captures self-reported consumption and not OTC sales.

### Independent variables

Independent variables or drivers for taking OTC drugs are grouped according to the Andersen model: predisposing, enabling and needs factors, which are presented and described in Table 1.

Firstly to be noted, as Cluster 5 accounts for a single country – Portugal (Vila et al., 2023), it was combined with the cluster closest to it, Cluster 6. In this way, there are two groups of clusters differing in their extent of OTC market liberalisation: ‘pharmacy retail only’ and ‘pharmacy and non-pharmacy retail’. Each group accounts for three clusters of countries which are listed in Table 1.

Secondly, the number of days people walk or cycle for at least 10 min, during the week, allowing for the possibility to stop by or small deviations to enter a pharmacy to get medicines serves as a proxy for pharmacy accessibility (Vogler et al., 2014).

### Analysis strategy

A descriptive analysis is performed first. Next, we estimate a logistic regression for three specifications: no country/cluster controls; cluster controls; and country controls. We also estimate the same model for each cluster. We present the odd ratios, the *p-value*, and 95% confidence interval. The estimation procedure accounts for potential heteroscedasticity and so standard errors are robust estimations.

Several tests are performed. To test the existence of multicollinearity we use a VIF test; to test the goodness-of-fit we estimate the area under the ROC curve, the Pearson statistic, and the specificity and sensitivity of the model. All the computations were performed in STATA 16.

## Findings

### Descriptive analysis

The descriptive analysis of the EHIS sample shows that of the 286,413 individuals, 33.6% of respondents had taken OTCs in the previous two weeks. The share of respondents using OTC drugs in each country is presented in Appendix Table A2. Additionally, Appendix Table A3 shows this percentage per cluster of countries. The cluster with the largest share of OTC consumption is Cluster 2, where 52.4% of respondents report taking OTC drugs, while Clusters 5 and 6 have the lowest percentages, about 22%. Additionally, Appendix Table A3 shows that the average of the percentage of respondents using OTC drugs in the group ‘Pharmacy retail only’ is 41.2% while in the group ‘Pharmacy and non-pharmacy retail’ it is 33.8%.

The descriptive results are presented in Table 2. Regarding these results, the following is worth highlighting: (i) there is a large percentage of people who walk for at least 10 min per day on week days and a large majority of people do not cycle; (ii) the majority of people report suffering from some level of pain; (iii) nearly 14% of people report unmet health care due to waiting and about 4% due to financial constraints; (iv) finally, nearly half of respondents are taking prescribed drugs.

**Table 2.** Statistical description for sample.

Variable label	% or mean^a^	Variable label	%^a^	Variable label	% or mean^a^
Predisposing factors		Enabling factors		Need factors	
male	46.1%	(Income quantil) Q1	16.96%	SAH_VGood	23.63%
age (mean)	53.6	Q2	18.67%	SAH_Good	42.73%
education (mean)	6.9	Q3	19.28%	SAH_Fair	24.96%
family_size (mean)	2.6	Q4	19.63%	SAH_Bad	7.06%
single	27.5%	Q5	19.23%	SAH_VeryBad	1.62%
married	53.2%	employed	46.98%	chronic_disease	47.5%
widow	11.1%	unemployed	4.95%	pain (no pain)	46.3
divorced	7.9%	retired	29.95%	unmet_waiting^b^	13.7%
cities	35.3%	student	7.38%	unmet_distance^b^	2.9%
rural	31.5%	other	10.19%	unmet_financial^b^	4.38%
towns	33.1%	walk _nrdays (none)	22.17%	bmi (mean)^c^	22.2
		(walk) 5 days	12.34%	rx_drugs^d^	49.5%
		(walk) 7 days	39.44%		
		bike_nrdays (none)	78.66%		
		(bike) 5 days	3.00%		
		(bike) 7 days	3.09%		
		(bike) 1or2 days	8.76%		

^a^ Units are the percentage or the mean of the responses given by individuals in the sample.

^b^ Unmet means unmet need for health care followed by the motive.

^c^ bmi – body mass index.

^d^ rx – prescribed.

The pairwise correlations between binary variables are contained in Table SM1 and those between non-binary variables in Supplemental Table SM2. In general, there are no strong correlations between variables. Worth mentioning, however, are the strong positive correlations between the following: reporting suffering from chronic diseases and being retired; taking prescribed drugs and reporting a less than good self-assessed health; taking prescribed drugs and being retired; unmet health needs due to distance and due to financial constraints; and finally, between unmet health needs due to distance and due to waiting time.

### Model estimation results

The results found for the VIF test, in Appendix Table A1, show that there is no multicollinearity.

Based on the model specification without country or cluster controls, the estimated area under the ROC curve is about 0.724; the Pearson-chi2 statistic has a *p-value* of .117; the estimated model correctly classifies about 70% of the cases, with a sensitivity of 39.4% and a specificity of 86.7%. Thus, it may be concluded that the estimated model fits the data well in general.

The logistic regression results are presented in Table 3, which shows the estimated odd ratios grouped according to Andersen model factors. The table shows three different model specifications: (i) no country nor cluster control, (ii) clusters control, and (iii) country controls. A summary of the estimated sign correlations is also available in Table 4.

**Table 3.** Results explaining OTC consumption.

Dependent variable: OTC – respondent uses OTC drugs (yes – takes value 1; no – takes value 0)												
	No country/cluster controls				Cluster controls				Country controls			
	OR	*P* > z	95%CI		OR	*P* > *z*	95%CI		OR	*P* > *z*	95%CI	
**Predisposing factors**												
male	0.585	0.000	0.575	0.595	0.594	0.000	0.584	0.605	0.611	0.000	0.599	0.622
age	0.994	0.000	0.994	0.995	0.995	0.000	0.994	0.996	0.996	0.000	0.995	0.997
education	1.029	0.000	1.027	1.030	1.027	0.000	1.025	1.028	1.021	0.000	1.020	1.023
family_size	0.963	0.000	0.956	0.970	0.969	0.000	0.961	0.976	0.951	0.000	0.943	0.958
single	1.054	0.006	1.015	1.093	1.020	0.289	0.983	1.059	0.998	0.903	0.960	1.037
married	1.066	0.000	1.033	1.100	1.054	0.001	1.021	1.088	1.051	0.003	1.017	1.086
divorced	1.156	0.000	1.111	1.203	1.097	0.000	1.053	1.142	1.061	0.005	1.018	1.105
cities	1.075	0.000	1.053	1.098	1.048	0.000	1.026	1.070	1.061	0.000	1.038	1.084
rural	1.048	0.000	1.026	1.071	1.043	0.000	1.020	1.065	0.935	0.000	0.915	0.957
**Enabling factors**												
Q1 (poorest)	0.883	0.000	0.858	0.909	0.902	0.000	0.876	0.928	0.852	0.000	0.827	0.878
Q2	0.867	0.000	0.844	0.891	0.900	0.000	0.875	0.925	0.884	0.000	0.859	0.909
Q3	0.938	0.000	0.914	0.962	0.981	0.152	0.956	1.007	0.966	0.013	0.941	0.993
Q4	0.958	0.001	0.934	0.982	0.993	0.615	0.968	1.019	0.995	0.713	0.969	1.022
employed	1.312	0.000	1.271	1.355	1.350	0.000	1.307	1.394	1.232	0.000	1.193	1.273
unemployed	0.920	0.001	0.876	0.966	1.007	0.773	0.959	1.058	0.979	0.425	0.931	1.031
retired	1.228	0.000	1.187	1.271	1.289	0.000	1.245	1.334	1.087	0.000	1.049	1.127
student	1.412	0.000	1.346	1.480	1.357	0.000	1.293	1.423	1.221	0.000	1.162	1.283
rx_drugs^a^	1.065	0.000	1.043	1.089	1.057	0.000	1.034	1.081	1.096	0.000	1.071	1.122
walk_nrdays	0.997	0.045	0.994	1.000	0.992	0.000	0.989	0.996	1.008	0.000	1.004	1.011
bike_nrdays	1.056	0.000	1.051	1.061	1.032	0.000	1.027	1.037	1.012	0.000	1.007	1.017
**Needs**												
SAH_Good	1.226	0.000	1.197	1.254	1.157	0.000	1.130	1.185	1.178	0.000	1.149	1.208
SAH_Fair	1.238	0.000	1.201	1.277	1.213	0.000	1.176	1.252	1.157	0.000	1.119	1.196
SAH_Bad	1.090	0.000	1.042	1.140	1.060	0.012	1.013	1.110	0.984	0.490	0.938	1.031
SAH_VeryBad	0.911	0.017	0.845	0.983	0.899	0.006	0.833	0.970	0.842	0.000	0.779	0.910
chronic_disease	1.219	0.000	1.192	1.246	1.175	0.000	1.149	1.202	1.192	0.000	1.164	1.221
pain	1.212	0.000	1.204	1.220	1.225	0.000	1.216	1.233	1.243	0.000	1.234	1.252
unmet_waiting^b^	1.186	0.000	1.156	1.216	1.185	0.000	1.155	1.216	1.221	0.000	1.189	1.254
unmet_distance^b^	1.026	0.336	0.974	1.081	1.022	0.433	0.969	1.077	1.008	0.780	0.955	1.063
unmet_financial^b^	1.212	0.000	1.163	1.263	1.269	0.000	1.216	1.323	1.222	0.000	1.171	1.276
bmi^c^	1.023	0.000	1.022	1.024	1.004	0.000	1.003	1.006	0.991	0.000	0.989	0.993
_cons	0.211	0.000	0.197	0.227	0.663	0.000	0.608	0.724	0.615	0.000	0.560	0.677
Cluster^d^	no				yes				no			
Cluster1					0.432	0.000	0.413	0.451				
Cluster3					0.645	0.000	0.615	0.676				
Cluster4					0.843	0.000	0.803	0.885				
Cluster5&6					0.292	0.000	0.279	0.306				
Cluster7					0.736	0.000	0.702	0.771				
Country controls	no				no				yes			
Number of observations	260,005				260,005				260,005			
Wald *χ*^2^(30)	18,166.62				23,850.37				31,435.05			
Prob > *χ*^2^	0.0000				0.000				0.000			
Pseudo *R*^2^	0.059				0.081				0.114			

Notes: OR – odd ratio; CI – confidence interval; *p*-value equal to ‘.000’ means *p*-value < .001.

^a^ rx – prescribed.

^b^ Unmet means unmet need for health care followed by the motive.

^c^ bmi – body mass index.

^d^ Cluster 1: Austria, Cyprus, Greece, Estonia, Germany, and Spain. Cluster 2: Finland and Luxembourg. Cluster 3: Belgium, Bulgaria, Latvia, Malta, and Slovakia. Cluster 4: Denmark, Hungary, Lithuania, and Sweden. Clusters 5 and 6: Croatia, Italy, Slovenia, Romania, and Portugal. Cluster 7: Czechia, Ireland, Netherlands, and Poland.

**Table 4.** Summary results explaining OTC consumption.

Dependent variable: OTC – respondent uses OTC drugs			
	Estimated correlation sign	Estimated correlation sign	Estimated correlation sign
**Predisposing factors**			
male	−	−	−
age	−	−	−
education	+	+	+
family_size	−	−	−
single	+	ns	ns
married	+	+	+
divorced	+	+	+
cities	+	+	+
rural	+	+	−
**Enabling factors**			
Q1 (poorest)	−	−	−
Q2	−	−	−
Q3	−	ns	−
Q4	−	ns	ns
employed	+	+	+
unemployed	−	ns	ns
retired	+	+	+
student	+	+	+
rx_drugs	+	+	+
walk_nrdays	−	−	+
bike_nrdays	+	+	+
**Needs**			
SAH_Good	+	+	+
SAH_Fair	+	+	+
SAH_Bad	+	+	ns
SAH_VeryBad	−	−	−
chronic_disease	+	+	+
pain	+	+	+
unmet_waiting	+	+	+
unmet_distance	+	ns	ns
unmet_financial	+	+	+
bmi	+	+	−
Cluster controls	No	Yes	No
Country controls	No	No	Yes

Note: sign ‘−’ means a negative correlation; sign ‘+’ means a positive correlation; ns − non-statistical significative.

Finally in Supplemental Table SM3, the results found for each cluster are presented.

The results presented in Table 3, and summarised in Table 4, show that there is a general consistency among the drivers for OTC consumption across the different model specifications which control for different individual effects.

Firstly, the set of predisposing factors tend be positively associated with OTC consumption, except for age and family size, which are negatively associated. On the other hand, being single has no statistical significance for the estimations accounting for cluster and country controls.

Secondly, concerning enabling factors, we found that in general they are associated with OTC consumption. As people’s incomes decrease, their propensity to buy OTC also decreases. However, for income quantile Q3 and Q4, we may find it is non-significant when controls for cluster or countries are considered. The variable capturing accessibility within walking distance reveals a negative association, although not very strong, which changes to positive when country-individual effects are included in the model. Finally, unemployment is a negative correlation in the model without individual controls, but it loses statistical significance in the other two model specifications.

Thirdly, concerning need factors, we have also found a general positive association, except for the health status ‘very bad’ which has a negative association in all model specifications and the health status ‘bad’ also has a negative association when cluster controls are included in the model. Finally, in all model specifications displayed in Table 3, we found no statistical significance for the unmet healthcare needs due to long-distance or transportation problems.

Fourthly, from the model specification with cluster controls, where Cluster 2 is taken as the reference category, the ranking which emerges from the estimated odd ratios coincides with the order that is obtained by the percentage of people self-reporting the use of OTCs in each cluster (Table A3, in Appendix), as expected. That is, the ranking from the cluster with the highest percentage of OTCs used by people to the one with the lowest is as follows: Cluster 2 (Finland and Luxembourg), Cluster 4 (Denmark, Hungary, Lithuania, and Sweden), Cluster 7 (Czechia, Ireland, Netherlands, and Poland), Cluster 3 (Belgium, Bulgaria, Latvia, Malta, and Slovakia), Cluster 1 (Austria, Cyprus, Greece, Estonia, Germany, and Spain), and lastly Clusters 5 and 6 (Croatia, Italy, Slovenia, Romania, and Portugal). It is noteworthy that Cluster 2 is in the ‘Pharmacy retail only’ group, while Clusters 5 and 6 is in the ‘Pharmacy and non-pharmacy retail’ group.

Next, we provide an overall view of the results found for each cluster of countries which is presented in Supplemental Table SM3. This table shows that there is some heterogeneity on the demand side of OTCs across clusters. However, there are clearly common factors. Across all clusters, being male and older decreases the likelihood of OTC consumption while higher education increases it. Moreover, suffering from a chronic disease or pain and being on a waiting list for medical treatment also increases the probability of consuming OTCs. Concerning the remaining factors, we find that they have statistical significance in some clusters but not in others. The worst fitting cluster is Cluster 2 (Luxembourg and Finland), which has the largest non-significant estimated odd ratios, while Cluster 3 (Belgium, Latvia, Bulgaria, Slovakia, and Malta) has the largest number of significant variables. Both clusters are from the same group of market regulation, meaning the ‘Pharmacy retail only’ group of countries.

## Discussion

Over the last few decades, the movement towards self-medication, the switch from prescription drugs to OTCs and the liberalisation of the pharmacy market have increased the consumption of OTC medicines in Europe. This work is aimed at finding the drivers for such consumption, considering the similarity of the regulatory framework across the countries studied. To accomplish this goal, we have estimated a logistic model for OTC consumption based on the Andersen model for medical services use (Andersen, 1968, 1981), on the empirical model for OTC drivers by Tavares et al. (2022), and on the market regulation clusters of countries proposed by Vila et al. (2023), using data from EHIS w3 for 26 European countries.

### Key findings

The first finding is the absence of a clear relationship between OTC market deregulation and OTC consumption across the EU countries included in the sample.

Secondly, the set of predisposing, enabling, and needs factors are drivers of OTC consumption in European countries. Even accounting for country and/or cluster controls, the significant factors do not change much. By taking this into account, we have found a general trend in European countries associated with the drivers of OTC consumption.

However, when clusters are considered isolated, we find some heterogeneity across the different clusters. This heterogeneity refers to the fact that there is a set of factors which are significant in some countries but not in others. This heterogeneity does not exist for a set of enabling factors (gender, age, and education) and need factors (suffering from chronic disease or pain and being on a waiting list for medical treatment). These results are quite relevant because, even when accounting for market regulations, people have identical factors influencing the decision to buy and consume OTC drugs.

### Interpretation

We start by commenting on the surprising finding that the consumption of OTC drugs is not strictly related to the level of OTC drugs market liberalisation. Countries such as those in Clusters 5 and 6, which comprises Portugal, Croatia, Italy, Romania, Slovenia and Croatia, benefit from a deregulated OTC drugs market but people are less likely to report consuming OTCs than people in the countries of Cluster 3 countries (Belgium, Bulgaria, Latvia, Slovakia, and Malta) where retailing is exclusive of pharmacies. In fact, on average, the group of countries with a stricter market regulation has an OTC consumption share of 41.2%, while the group of countries with wider market liberalisation has an OTC consumption share of 37.6%. The case of Finland and Luxembourg is a good example of this controversial result: in these countries, the market regulation is tighter and pharmacy chains (Pisek, 2017; Vogler et al., 2014) are not allowed. Despite this, more than half of the respondents consume OTC drugs.

The drivers of OTC consumption are thus as follows. Firstly, we found that there is some heterogeneity across clusters. In Cluster 2 (Luxembourg and Finland), for instance, the likelihood of finding significant variables was lower, while the opposite was true for Cluster 3 (Belgium, Latvia, Bulgaria, Slovakia, and Malta), which was more likely to fit the estimation model well. This could be a result of the sub-sample size, although this justification would not be applicable to Cluster 7 (Czechia, Ireland, Netherlands, and Poland) which accounts for a larger sub-sample, although several variables are not statistically significant. This cross-cluster heterogeneity may result from cultural differences or from health system differences, which impact the way people decide to buy and consume OTCs.

Cultural differences have long been recognised as factors influencing individuals’ decisions (Abel, 2008; Caldwell & Caldwell, 1991; Mackenbach, 2014; Roudijk et al., 2017) and their health. It could be that in countries with a stronger risk avoidance, people will tend to consume fewer OTCs because of their potential adverse effects; but it could also be that in countries with a stronger orientation towards the present, people may have a greater propensity for consuming OTCs to solve some minor illnesses quickly.

Another difference that may cause heterogeneity across countries is the difference of perceived, reported pain, and the tendency to alleviate it with painkillers. Since the most commonly used OTC drugs are pain relievers (RxList, 2024), there may be differences in OTC consumption across countries arising from these differences. Finally, institutional differences explain heterogeneity. Health system coverage of prescribed drugs differ across countries. In health systems with more wide coverage on pharmaceuticals, there may be a less of a reliance on the use of OTC drugs as people may prefer to access prescribed drugs that may substitute those OTC drugs. Differences in the labour market also contribute to heterogeneity. The legal differences and cost-share of work absenteeism may induce people to take OTCs to minimise the number of work-absent days. All these reasons are topics for future research to better understand differences of OTC market regulation across clusters of countries.

Despite this heterogeneity, there are common drivers across countries: age, gender, education, suffering from long-lasting problems or pain, and being on a waiting list for health care. The results found for the predisposing factors age, gender and education were also found in other studies, despite some slight differences. As people get older in the UK, Bulgaria, Romania, and South Korea (Cho & Lee, 2013; Green et al., 2016; Porteous et al., 2015; Vogler et al., 2015), they are more likely to consume OTCs, while in countries like Portugal, Austria, Spain, and China (Chang et al., 2017; Daban et al., 2010; Jiménez-Rubio & Hernández-Quevedo, 2010; Mayer & Österle, 2015; Qin et al., 2022; Tavares et al., 2022), the opposite is expected to happen, as we found. This relationship may not be linear and depends on the strength of two effects.

On the one hand, younger and active people are more pressured to be healthy and, on the other, older people may feel less healthy and seek out faster solutions. We have found a negative association in all model specifications so perhaps the stronger effect comes from the members of the younger share of the population who wish to prevent inactive days due to ill-health.

Some empirical results found that there was a higher probability of buying OTC drugs among men in countries like South Korea (Cho & Lee, 2013). However, in Europe, we find that women are more likely to consume OTCs. This is a general result found for European countries. One possible reason for this fact was proposed by Tavares et al. (2022). Women have learned to make use of pharmacies and have more experience doing so in order to obtain contraceptives, including the emergency contraceptive pill. This has improved their readiness to use pharmacies to buy other OTC drugs. Another reason that could explain the higher likelihood of the use of OTC by women is that they tend to be family carers, which enforces self-responsibility and others care concerning health. In line with gender, higher education is associated with a higher likelihood of consuming OTCs across European countries. Higher education ensures individual resources (cognitive, communicative, informative, relational) which contribute to empowerment over health decisions.

Regarding the enabling factors – suffering from a chronic disease or pain and being on a waiting list to get health care – some studies also confirm the findings reported here. First, concerning chronic diseases and/or pain. Identical results were previously found in Portugal, South Korea, Austria, China, Spain, and Eastern European countries (Chang et al., 2017; Daban et al., 2010; Jiménez-Rubio & Hernández-Quevedo, 2010; Mayer & Österle, 2015; Tavares et al., 2022; Vogler et al., 2015). The most common OTC drugs are painkillers, medicines for heartburn and indigestion, laxatives and antidiarrheal drugs, cough medicine, and antihistamines. Any of these conditions are easily associated with chronic disease and pain, so it is very likely that people will seek out pharmacies in the first place instead of medical care.

Secondly, we found that waiting for health care contributes to the likelihood of taking OTCs. In fact, difficult access to health care is a driver of OTC consumption and self-medication around the world (Shaghaghi et al. 2014). Even though waiting for health care may be perceived differently according to health systems and excessive waiting time may be defined in different ways, it may happen that while waiting, people may have the medical and pharmaceutical advice to deal with mild symptoms of an illness. Very often waiting may be used as an observational period to assess the evolution of the health condition. In other situations, waiting for health care on a list requires management of the symptoms until the visit to the health care service can take place On the other hand, waiting too long and taking OTC without medical supervision increases the risk of self-medication and it can also increase the burden on the health system due to more expensive treatments. Therefore, primary care plays an important role in moderating this waiting time and OTC use to prevent worse health and financial outcomes.

Lastly, the results obtained for the whole sample, show that the set of predisposing, enabling and need factors play a role in the consumption of OTCs across European countries, even when controlling for individual country effects. Here we will discuss two broad drivers: accessibility and health care. First, findings indicate that walking and cycling is associated with a slight increase in the consumption of OTC drugs, after taking countries as controls and within some clusters of countries. This may occur because it becomes easy to make a stop or take a small detour to access a pharmacy or any other drug retailer. On the other hand, it is easier to obtain OTCs in urban areas than in rural areas, as expected from previous results (Cohen et al., 2013), which showed that deregulation of OTC markets tended to benefit urban areas. This finding is also likely to occur more often, the larger emphasis being placed on the creation and implementation of the ‘15-minute city’, which reduces distance and effort required to reach pharmacies and other retail shops.

The second influence of the consumption of OTCs is related to health care. On the one hand, we found a positive relationship between the consumption of OTCs and the consumption of prescribed drugs. This finding is particularly interesting in a context of OTC market deregulation. Despite the possibility for people to buy OTCs in non-pharmacy retailers, the fact that people need to go to a pharmacy to get prescription drugs (and about half of the respondents take prescription drugs) may allow for the inference that market liberalisation has a small impact on people’s decision. Nowadays pharmacies offer a wide range of services to maintain revenues (Anell 2005; Kanavos et al., 2011; Rutter, 2015; Vogler et al., 2014; Wooyong et al., 2022), including selling OTCs, and people may not be so attracted by OTC non-pharmacy retailers.

On the other hand, we found a negative relationship, expressing a substitution effect, between the consumption of OTCs and the unmet healthcare needs due to financial household constraints, as previously reported to Portugal (Tavares et al., 2022). On the one hand, it may be that there is a perception that OTCs are cheaper than prescribed drugs or health care appointments (Chang et al., 2017). On the other hand, this is a red light in a set of countries which are expected to ensure universal health care coverage. Not only do these people face financial constraints to access health care, but they also risk the inappropriate use of OTCs and the worsening of their ill-health condition.

### Strengths and limitations

The major strength and contribution of this work comes from the analysis of factors associated with the consumption of OTCs in a large set of European countries while taking into consideration of OTC market regulation.

The limitations of our work derive from the fact that we have not considered relevant contextual factors such as health system characteristics and cultural factors. While the health system characteristics may directly influence the number of prescribed drugs and access to other health care services, cultural factors influence the individual decisions of people, and it may be the case that countries with stronger risk avoidance show less of a tendency to buy OTCs. However, these characteristics are captured in the individual country effects. Future research may investigate the association of specific characteristics of the health system and of the cultural context with the decision to consume OTCs more extensively. The difference in the average distance to the nearest pharmacy indicated by Vogler et al. (2014) was controlled by two variables: number of days walking or cycling a minimum distance of 10 min to get to places. However, these two variables may be subject to criticism as they do not directly capture the average distance to pharmacies, but is used as a proxy for accessibility under the concept of the ‘15-minute city’. On the other hand, pharmacy accessibility may also include other indicators such as opening hours, services provided, and variety of drugs on offer. So, pharmacy accessibility is only partially controlled for and may be prone to some criticism, which may justify future research to understand which are the most important accessibility factors for consumers.

Another potential limitation concerns definition of the outcome variable describing taking OTC. This variable is self-reported which means it may be subject to some bias. On the one hand, if doctors suggest or prescribe OTC, then it may be that this variable is overestimated. On the other, it may be that people are not fully aware of what OTCs are and under-report their consumption. Overall, we believe there is some fair averaging up between the two situations.

Finally, it is worth mentioning that this type of study does not allow for causality conclusions, all the results obtained are to be interpreted as correlations obtained across individuals in one period of time.

## Conclusions

This study shows that there is some heterogeneity across countries and clusters of countries with similar OTC retail market regulation. On the one hand, market regulation is not associated with the level of OTC consumption in each country. On the other hand, despite some similar predisposing, enabling, and need factors associated with OTC use, there are also some slight differences across cluster of countries with identical market liberalisation.

The major policy implication of this work concerns the construction of the European Health Union. Despite the common European Medicines Agency, there are differences between member states, such as OTC retail market regulation, which may contribute to inequalities in OTC access and health. The second major policy implication relates to the negative relationship between OTC drugs and access to health care due to financial constraints which raises equity concerns for universal health coverage of European health systems.

## Supplementary Material

Supplemental Material

## Data Availability

The author does not have permission to share the data on EHIS. Interested parties may apply to access it through Eurostat. The remaining data are publicly available.

## Appendix

**Table A1.** VIF test.

Variable	VIF	1/VIF		Variable	VIF	1/VIF
single	3.97	0.25		pain	1.46	0.68
married	3.61	0.28		Q4	1.45	0.69
employed	3.54	0.28		family_size	1.38	0.72
retired	3.43	0.29		cities	1.37	0.73
SAH_Fair	2.43	0.41		rural	1.35	0.74
age	2.18	0.46		SAH_VeryBad	1.24	0.81
student	1.95	0.51		education	1.21	0.82
SAH_Bad	1.81	0.55		unmet_waiting^a^	1.17	0.85
divorced	1.80	0.56		unmet_distance^a^	1.14	0.88
SAH_Good	1.79	0.56		bmi	1.12	0.89
chronic_diseases	1.75	0.57		unmet_financial^a^	1.10	0.91
rx_drugs	1.65	0.60		male	1.10	0.91
Q1(poorest)	1.62	0.62		walk_nrdays	1.05	0.95
Q2	1.56	0.64		bike_nrdays	1.03	0.97
unemployed	1.53	0.66				
Q3	1.50	0.67		Mean VIF	1.78	

^a^ Unmet means unmet need for health care followed by the motive.

**Table A2.** Percentage of people self-reporting the use of OTC per country.

country	countrycode	OTC (%)	country	countrycode	OTC (%)
Austria	AT	39.4	Ireland	IE	30.1
Belgium	BE	27.1	Italy	IT	18.3
Bulgaria	BG	36.2	Lituania	LT	59.2
Cyprus	CY	56.3	Luxembourg	LU	37.0
Czechia	CZ	53.2	Latvia	LV	49.0
Germany	DE	39.3	Malta	MT	35.6
Denmark	DK	56.3	Netherlands	NL	41.6
Estonia	EE	53.8	Poland	PL	39.7
Greece	EL	20.8	Portugal	PT	22.4
Spain	ES	13.7	Romania	RO	20.6
Finland	FI	63.5	Sweden	SE	39.1
Croatia	HR	34.9	Slovenia	SI	35.4
Hungary	HU	53.9	Slovakia	SK	54.4

**Table A3.** Percentage of people self-reporting the use of OTC per cluster.

	Cluster^a^	*N*	OTC (%)	Cluster average OTC (%)
Pharmacy retail only	1	79 696	32.5	
	2	10 755	52.4	41.2
	3	33 157	38.8	
Pharmacy and non-pharmacy retail	4	26 912	49.9	
	5	14 617	22.4	
	6	77 509	22.1	33.8
	7	43 767	40.9	

^a^ Cluster 1: Austria, Cyprus, Greece, Estonia, Germany, and Spain. Cluster 2: Finland and Luxembourg. Cluster 3: Belgium, Bulgaria, Latvia, Malta, and Slovakia. Cluster 4: Denmark, Hungary, Lithuania, and Sweden. Clusters 5 and 6: Croatia, Italy, Slovenia, Romania, and Portugal. Cluster 7: Czechia, Ireland, Netherlands, and Poland
