# Supplementary material for: Over-the-counter drug consumption and related factors, evidence from the European Health Interview Survey
Source: J Pharm Policy Pract. 2025 Feb 3;18(1):2455068. doi: 10.1080/20523211.2025.2455068 (PMC11792145; doi:10.1080/20523211.2025.2455068)
Supplement: Supplemental Material [file JPPP_A_2455068_SM6932.docx]

**Supplemental Material**

Table SM1: Pairwise correlations across binary variables

|  | | OTC | | male | | single | | married | | divorced | | cities | | rural | | Q1(poorest) | | Q2 | | Q3 | | | Q4 | | Q5 | |
| --- | --- | --- | --- | --- | --- | --- | --- | --- | --- | --- | --- | --- | --- | --- | --- | --- | --- | --- | --- | --- | --- | --- | --- | --- | --- | --- |
| OTC | | 1.000 | |  | |  | |  | |  | |  | |  | |  | |  | |  | | |  | |  | |
| male | | -0.203 | | 1.000 | |  | |  | |  | |  | |  | |  | |  | |  | | |  | |  | |
| single | | -0.059 | | 0.140 | | 1.000 | |  | |  | |  | |  | |  | |  | |  | | |  | |  | |
| married | | 0.005 | | 0.107 | | -1.000 | | 1.000 | |  | |  | |  | |  | |  | |  | | |  | |  | |
| divorced | | 0.090 | | -0.102 | | -1.000 | | -1.000 | | 1.000 | |  | |  | |  | |  | |  | | |  | |  | |
| cities | | 0.054 | | -0.020 | | 0.079 | | -0.081 | | 0.090 | | 1.000 | |  | |  | |  | |  | | |  | |  | |
| rural | | -0.017 | | 0.015 | | -0.052 | | 0.046 | | -0.094 | | -1.000 | | 1.000 | |  | |  | |  | | |  | |  | |
| Q1 | | -0.029 | | -0.092 | | 0.099 | | -0.253 | | 0.144 | | -0.081 | | 0.096 | | 1.000 | |  | |  | | |  | |  | |
| Q2 | | -0.033 | | -0.064 | | -0.043 | | -0.054 | | 0.026 | | -0.068 | | 0.063 | | -1.000 | | 1.000 | |  | | |  | |  | |
| Q3 | | 0.000 | | 0.022 | | -0.033 | | 0.058 | | -0.017 | | -0.031 | | 0.017 | | -1.000 | | -1.000 | | 1.000 | | |  | |  | |
| Q4 | | 0.010 | | 0.047 | | -0.002 | | 0.080 | | -0.054 | | 0.042 | | -0.075 | | -1.000 | | -1.000 | | -1.000 | | | 1.000 | |  | |
| Q5 | | 0.035 | | 0.085 | | -0.017 | | 0.127 | | -0.064 | | 0.153 | | -0.172 | | -1.000 | | -1.000 | | -1.000 | | | -1.000 | | 1.000 | |
| employed | | 0.059 | | 0.174 | | 0.107 | | 0.144 | | 0.096 | | 0.038 | | -0.045 | | -0.334 | | -0.225 | | 0.001 | | | 0.190 | | 0.335 | |
| unemployed | | -0.104 | | 0.026 | | 0.250 | | -0.163 | | 0.092 | | 0.004 | | -0.020 | | 0.343 | | 0.077 | | -0.078 | | | -0.183 | | -0.286 | |
| retired | | 0.024 | | -0.014 | | -0.593 | | 0.086 | | -0.008 | | -0.049 | | 0.060 | | 0.043 | | 0.187 | | 0.057 | | | -0.082 | | -0.181 | |
| student | | -0.085 | | 0.020 | | 0.771 | | -0.656 | | -0.426 | | 0.048 | | -0.028 | | 0.147 | | -0.009 | | -0.049 | | | -0.065 | | -0.113 | |
| rx_drugs | | 0.176 | | -0.143 | | -0.394 | | 0.101 | | 0.093 | | 0.000 | | 0.004 | | 0.061 | | 0.098 | | 0.018 | | | -0.054 | | -0.093 | |
| SAH_VGood | | -0.121 | | 0.080 | | 0.349 | | -0.123 | | -0.111 | | 0.047 | | -0.044 | | -0.094 | | -0.099 | | -0.033 | | | 0.051 | | 0.138 | |
| SAH_Good | | 0.029 | | 0.026 | | -0.002 | | 0.092 | | 0.013 | | 0.025 | | -0.061 | | -0.104 | | -0.077 | | 0.012 | | | 0.069 | | 0.108 | |
| SAH_Fair | | 0.116 | | -0.087 | | -0.290 | | 0.050 | | 0.070 | | -0.055 | | 0.070 | | 0.110 | | 0.111 | | 0.027 | | | -0.072 | | -0.172 | |
| SAH_Bad | | 0.080 | | -0.084 | | -0.254 | | -0.051 | | 0.079 | | -0.047 | | 0.069 | | 0.182 | | 0.133 | | -0.007 | | | -0.119 | | -0.229 | |
| SAH_VBad | | 0.038 | | -0.074 | | -0.209 | | -0.076 | | 0.031 | | -0.035 | | 0.059 | | 0.166 | | 0.117 | | -0.003 | | | -0.127 | | -0.208 | |
| chronic_disease | | 0.176 | | -0.106 | | -0.324 | | 0.054 | | 0.088 | | 0.029 | | 0.017 | | 0.077 | | 0.104 | | 0.011 | | | -0.061 | | -0.110 | |
| unmet_waiting | | 0.157 | | -0.118 | | -0.075 | | 0.012 | | 0.068 | | 0.030 | | -0.029 | | 0.041 | | 0.036 | | 0.000 | | | -0.034 | | -0.054 | |
| unmet_distance | | 0.085 | | -0.104 | | -0.081 | | -0.062 | | 0.056 | | -0.094 | | 0.090 | | 0.166 | | 0.079 | | -0.024 | | | -0.102 | | -0.153 | |
| unmet_financial | | 0.127 | | -0.121 | | -0.057 | | -0.066 | | 0.132 | | -0.003 | | 0.017 | | 0.230 | | 0.085 | | -0.030 | | | -0.131 | | -0.223 | |
| (cont) | |  | |  | |  | |  | |  | |  | |  | |  | |  | |  | | |  | |  | |
|  | employed | | unemployed | | retired | | student | | rx_  drugs | | SAH_  VGood | | SAH_  Good | | SAH_  Fair | | SAH_  Bad | | SAH_  VBad | | chronic  diseases | unmet_  waiting | | unmet_  distance | |  |
| employed | 1.000 | |  | |  | |  | |  | |  | |  | |  | |  | |  | |  |  | |  | |  |
| unemployed | -1.000 | | 1.000 | |  | |  | |  | |  | |  | |  | |  | |  | |  |  | |  | |  |
| retired | -1.000 | | -1.000 | | 1.000 | |  | |  | |  | |  | |  | |  | |  | |  |  | |  | |  |
| student | -1.000 | | -1.000 | | -1.000 | | 1.000 | |  | |  | |  | |  | |  | |  | |  |  | |  | |  |
| rx_drugs | -0.396 | | -0.142 | | 0.567 | | -0.352 | | 1.000 | |  | |  | |  | |  | |  | |  |  | |  | |  |
| SAH_VGood | 0.289 | | 0.033 | | -0.503 | | 0.362 | | -0.565 | | 1.000 | |  | |  | |  | |  | |  |  | |  | |  |
| SAH_Good | 0.226 | | 0.005 | | -0.182 | | -0.070 | | -0.108 | | -1.000 | | 1.000 | |  | |  | |  | |  |  | |  | |  |
| SAH_Fair | -0.314 | | -0.019 | | 0.399 | | -0.323 | | 0.506 | | -1.000 | | -1.000 | | 1.000 | |  | |  | |  |  | |  | |  |
| SAH_Bad | -0.459 | | -0.010 | | 0.378 | | -0.334 | | 0.566 | | -1.000 | | -1.000 | | -1.000 | | 1.000 | |  | |  |  | |  | |  |
| SAH_VBad | -0.480 | | -0.073 | | 0.317 | | -0.319 | | 0.497 | | -1.000 | | -1.000 | | -1.000 | | -1.000 | | 1.000 | |  |  | |  | |  |
| chronic_disease | -0.359 | | -0.107 | | 0.483 | | -0.325 | | 0.727 | | -0.657 | | -0.222 | | 0.568 | | 0.742 | | 0.666 | | 1.000 |  | |  | |  |
| unmet_waiting^a^ | -0.050 | | 0.015 | | 0.048 | | -0.108 | | 0.286 | | -0.273 | | -0.088 | | 0.199 | | 0.274 | | 0.240 | | 0.305 | 1.000 | |  | |  |
| unmet_distance^a^ | -0.209 | | 0.056 | | 0.139 | | -0.121 | | 0.255 | | -0.280 | | -0.177 | | 0.138 | | 0.341 | | 0.355 | | 0.279 | 0.695 | | 1.000 | |  |
| unmet_financial^a^ | -0.136 | | 0.157 | | 0.044 | | -0.142 | | 0.230 | | -0.270 | | -0.195 | | 0.198 | | 0.324 | | 0.315 | | 0.289 | 0.486 | | 0.545 | |  |

Note: ^a^ unmet means unmet need for health care followed by the motive.

Table SM2: Pairwise correlations across non-binary variables

|  | OTC | age | education | family_size | pain | walk_nrdays | walk_nrdays |
| --- | --- | --- | --- | --- | --- | --- | --- |
| age | 0.007 | 1.000 |  |  |  |  |  |
|  | 0.000 |  |  |  |  |  |  |
| education | 0.114 | -0.178 | 1.000 |  |  |  |  |
|  | 0.000 | 0.000 |  |  |  |  |  |
| family_size | -0.049 | -0.324 | 0.012 | 1.000 |  |  |  |
|  | 0.000 | 0.000 | 0.000 |  |  |  |  |
| pain | 0.160 | 0.223 | -0.077 | -0.138 | 1.000 |  |  |
|  | 0.000 | 0.000 | 0.000 | 0.000 |  |  |  |
| walk_nrdays | 0.025 | -0.071 | 0.071 | 0.004 | -0.122 | 1.000 |  |
|  | 0.000 | 0.000 | 0.000 | 0.041 | 0.000 |  |  |
| walk_nrdays | 0.025 | -0.071 | 0.071 | 0.004 | -0.122 | 1.000 | 1.000 |
|  | 0.000 | 0.000 | 0.000 | 0.041 | 0.000 | 0.000 |  |
| bmi | 0.136 | 0.139 | 0.101 | -0.086 | 0.107 | 0.033 | 0.033 |
|  | 0.000 | 0.000 | 0.000 | 0.000 | 0.000 | 0.000 | 0.000 |

Table SM3: Results explaining OTC consumption for individual clusters

| \| Dependent variable: OTC – respondent uses OTC drugs \| \| \| \| \| \| \|  \|  \| \|  \|  \| \|  \|  \| \|  \|  \| \| \| --- \| --- \| --- \| --- \| --- \| --- \| --- \| --- \| --- \| --- \| --- \| --- \| --- \| --- \| --- \| --- \| --- \| --- \| --- \| \|  \|  \| Cluster 1^a^ (32.5%)^b^ \| \|  \| Cluster 2^a^ (52.4%)^b^ \| \|  \| Cluster 3^a^ (38.8%)^b^ \| \|  \| Cluster 4^a^ (49.9%)^b^ \| \|  \| Cluster 5&6^a^ (22.2%)^b^ \| \|  \| Cluster 7^a^ (40.9%)^b^ \| \| \|  \|  \| OR \| P>z \|  \| OR \| P>z \|  \| OR \| P>z \|  \| OR \| P>z \|  \| OR \| P>z \|  \| OR \| P>z \| \| Predisposing Factors \|  \|  \|  \|  \|  \|  \|  \|  \|  \|  \|  \|  \|  \|  \|  \|  \|  \|  \| \| male \|  \| 0.606 \| 0.000 \|  \| 0.553 \| 0.000 \|  \| 0.573 \| 0.000 \|  \| 0.581 \| 0.000 \|  \| 0.690 \| 0.000 \|  \| 0.563 \| 0.000 \| \| age \|  \| 0.996 \| 0.000 \|  \| 0.989 \| 0.000 \|  \| 0.996 \| 0.001 \|  \| 0.997 \| 0.001 \|  \| 0.997 \| 0.000 \|  \| 0.997 \| 0.000 \| \| education \|  \| 1.019 \| 0.000 \|  \| 1.023 \| 0.000 \|  \| 1.027 \| 0.000 \|  \| 1.019 \| 0.000 \|  \| 1.019 \| 0.000 \|  \| 1.020 \| 0.000 \| \| family_size \|  \| 0.954 \| 0.000 \|  \| 0.992 \| 0.707 \|  \| 0.916 \| 0.000 \|  \| 0.945 \| 0.000 \|  \| 0.969 \| 0.000 \|  \| 0.946 \| 0.000 \| \| single \|  \| 0.977 \| 0.518 \|  \| 1.024 \| 0.810 \|  \| 1.005 \| 0.926 \|  \| 1.114 \| 0.077 \|  \| 0.983 \| 0.654 \|  \| 1.002 \| 0.974 \| \| married \|  \| 1.070 \| 0.029 \|  \| 1.131 \| 0.175 \|  \| 1.148 \| 0.003 \|  \| 1.108 \| 0.056 \|  \| 0.952 \| 0.132 \|  \| 1.073 \| 0.079 \| \| divorced \|  \| 0.994 \| 0.877 \|  \| 1.230 \| 0.070 \|  \| 1.084 \| 0.163 \|  \| 1.138 \| 0.035 \|  \| 1.040 \| 0.374 \|  \| 1.124 \| 0.025 \| \| cities \|  \| 1.081 \| 0.000 \|  \| 1.056 \| 0.338 \|  \| 1.124 \| 0.000 \|  \| 0.990 \| 0.765 \|  \| 1.045 \| 0.043 \|  \| 1.059 \| 0.044 \| \| rural \|  \| 0.985 \| 0.504 \|  \| 1.088 \| 0.118 \|  \| 0.907 \| 0.004 \|  \| 0.852 \| 0.000 \|  \| 0.922 \| 0.000 \|  \| 0.932 \| 0.013 \| \| Enabling Factors \|  \|  \|  \|  \|  \|  \|  \|  \|  \|  \|  \|  \|  \|  \|  \|  \|  \|  \| \| Q1 (poorest) \|  \| 0.866 \| 0.000 \|  \| 0.988 \| 0.865 \|  \| 0.640 \| 0.000 \|  \| 0.908 \| 0.038 \|  \| 0.794 \| 0.000 \|  \| 0.912 \| 0.011 \| \| Q2 \|  \| 0.877 \| 0.000 \|  \| 1.090 \| 0.203 \|  \| 0.754 \| 0.000 \|  \| 0.906 \| 0.021 \|  \| 0.821 \| 0.000 \|  \| 0.959 \| 0.234 \| \| Q3 \|  \| 0.927 \| 0.004 \|  \| 1.156 \| 0.035 \|  \| 0.831 \| 0.000 \|  \| 1.119 \| 0.006 \|  \| 0.927 \| 0.005 \|  \| 1.014 \| 0.682 \| \| Q4 \|  \| 0.953 \| 0.060 \|  \| 1.093 \| 0.183 \|  \| 0.880 \| 0.001 \|  \| 1.081 \| 0.049 \|  \| 0.998 \| 0.931 \|  \| 1.028 \| 0.407 \| \| employed \|  \| 1.128 \| 0.000 \|  \| 1.113 \| 0.233 \|  \| 1.304 \| 0.000 \|  \| 1.151 \| 0.006 \|  \| 1.356 \| 0.000 \|  \| 1.140 \| 0.001 \| \| unemployed \|  \| 0.911 \| 0.068 \|  \| 0.904 \| 0.478 \|  \| 1.048 \| 0.492 \|  \| 0.827 \| 0.028 \|  \| 1.101 \| 0.035 \|  \| 1.031 \| 0.706 \| \| retired \|  \| 1.002 \| 0.962 \|  \| 1.007 \| 0.944 \|  \| 1.244 \| 0.000 \|  \| 1.102 \| 0.084 \|  \| 1.100 \| 0.007 \|  \| 1.048 \| 0.274 \| \| Student \|  \| 1.170 \| 0.002 \|  \| 1.456 \| 0.002 \|  \| 1.323 \| 0.000 \|  \| 1.207 \| 0.012 \|  \| 1.284 \| 0.000 \|  \| 1.005 \| 0.935 \| \| rx_drugs^c^ \|  \| 0.924 \| 0.000 \|  \| 1.331 \| 0.000 \|  \| 1.151 \| 0.000 \|  \| 1.206 \| 0.000 \|  \| 1.213 \| 0.000 \|  \| 1.022 \| 0.471 \| \| walk_nrdays \|  \| 1.005 \| 0.144 \|  \| 1.017 \| 0.076 \|  \| 1.018 \| 0.001 \|  \| 1.012 \| 0.027 \|  \| 1.003 \| 0.403 \|  \| 1.010 \| 0.025 \| \| bike_nrdays \|  \| 1.018 \| 0.000 \|  \| 1.015 \| 0.284 \|  \| 1.019 \| 0.020 \|  \| 1.000 \| 0.954 \|  \| 1.016 \| 0.007 \|  \| 1.007 \| 0.271 \| \| Needs \|  \|  \|  \|  \|  \|  \|  \|  \|  \|  \|  \|  \|  \|  \|  \|  \|  \|  \| \| SAH_Good \|  \| 1.129 \| 0.000 \|  \| 1.024 \| 0.717 \|  \| 1.276 \| 0.000 \|  \| 1.130 \| 0.001 \|  \| 1.316 \| 0.000 \|  \| 1.148 \| 0.000 \| \| SAH_Fair \|  \| 1.060 \| 0.061 \|  \| 0.970 \| 0.712 \|  \| 1.346 \| 0.000 \|  \| 1.109 \| 0.035 \|  \| 1.241 \| 0.000 \|  \| 1.213 \| 0.000 \| \| SAH_Bad \|  \| 0.948 \| 0.241 \|  \| 0.694 \| 0.005 \|  \| 1.237 \| 0.001 \|  \| 1.029 \| 0.690 \|  \| 0.977 \| 0.635 \|  \| 0.977 \| 0.702 \| \| SAH_VeryBad \|  \| 0.834 \| 0.020 \|  \| 0.535 \| 0.032 \|  \| 1.087 \| 0.424 \|  \| 1.000 \| 0.997 \|  \| 0.789 \| 0.002 \|  \| 0.792 \| 0.022 \| \| chronic_disease \|  \| 1.244 \| 0.000 \|  \| 1.153 \| 0.011 \|  \| 1.325 \| 0.000 \|  \| 1.234 \| 0.000 \|  \| 1.063 \| 0.012 \|  \| 1.232 \| 0.000 \| \| pain \|  \| 1.216 \| 0.000 \|  \| 1.318 \| 0.000 \|  \| 1.263 \| 0.000 \|  \| 1.236 \| 0.000 \|  \| 1.249 \| 0.000 \|  \| 1.269 \| 0.000 \| \| unmet_waiting^d^ \|  \| 1.199 \| 0.000 \|  \| 1.345 \| 0.000 \|  \| 1.267 \| 0.000 \|  \| 1.284 \| 0.000 \|  \| 1.194 \| 0.000 \|  \| 1.238 \| 0.000 \| \| unmet_distance^d^ \|  \| 1.183 \| 0.003 \|  \| 0.826 \| 0.185 \|  \| 0.969 \| 0.715 \|  \| 1.153 \| 0.138 \|  \| 0.945 \| 0.246 \|  \| 0.937 \| 0.308 \| \| unmet_financial^d^ \|  \| 1.142 \| 0.004 \|  \| 1.093 \| 0.388 \|  \| 1.271 \| 0.000 \|  \| 1.139 \| 0.110 \|  \| 1.361 \| 0.000 \|  \| 1.107 \| 0.063 \| \| bmi^e^ \|  \| 0.990 \| 0.000 \|  \| 0.994 \| 0.203 \|  \| 0.985 \| 0.000 \|  \| 0.996 \| 0.227 \|  \| 0.990 \| 0.000 \|  \| 0.993 \| 0.003 \| \| country^f^ \|  \|  \|  \|  \|  \|  \|  \|  \|  \|  \|  \|  \|  \|  \|  \|  \|  \|  \| \|  \| CY \| 2.53 \| 0.00 \| LU \| 0.24 \| 0.00 \| BG \| 1.73 \| 0.00 \| HU \| 0.86 \| 0.00 \| IT \| 0.36 \| 0.00 \| IE \| 0.40 \| 0.00 \| \|  \| DE \| 0.86 \| 0.00 \| FI \| 1.00 \|  \| LV \| 2.45 \| 0.00 \| LT \| 1.00 \| 0.98 \| PT \| 0.45 \| 0.00 \| NL \| 0.56 \| 0.00 \| \|  \| EE \| 1.55 \| 0.00 \|  \|  \|  \| MT \| 2.02 \| 0.00 \| SE \| 0.39 \| 0.00 \| RO \| 0.55 \| 0.00 \| PL \| 0.64 \| 0.00 \| \|  \| EL \| 0.45 \| 0.00 \|  \|  \|  \| SK \| 3.21 \| 0.00 \| DK \| 1.00 \|  \| SI \| 0.81 \| 0.00 \| CZ \| 1.00 \|  \| \|  \| ES \| 0.26 \| 0.00 \|  \|  \|  \| BE \| 1.00 \|  \|  \|  \|  \| HR \| 1.00 \|  \|  \|  \|  \| \|  \| AU \| 1.00 \|  \|  \|  \|  \|  \|  \|  \|  \|  \|  \|  \|  \|  \|  \|  \|  \| \| _cons \|  \| 0.800 \| 0.010 \|  \| 1.885 \| 0.005 \|  \| 0.351 \| 0.000 \|  \| 1.061 \| 0.665 \|  \| 0.494 \| 0.000 \|  \| 1.154 \| 0.198 \| \| Number of obs \|  \| 75965 \|  \|  \| 9688 \|  \|  \| 30376 \|  \|  \| 25217 \|  \|  \| 82727 \|  \|  \| 36032 \|  \| \| Wald chi2(35) \|  \| 8673.90 \|  \|  \| 1350.66 \|  \|  \| 3173.46 \|  \|  \| 2173.95 \|  \|  \| 4959.10 \|  \|  \| 2743.55 \|  \| \| Prob > chi2 \|  \| 0.000 \|  \|  \| 0.000 \|  \|  \| 0.000 \|  \|  \| 0.000 \|  \|  \| 0.000 \|  \|  \| 0.000 \|  \| \| Pseudo R2 \|  \| 0.110 \|  \|  \| 0.124 \|  \|  \| 0.092 \|  \|  \| 0.071 \|  \|  \| 0.058 \|  \|  \| 0.062 \|  \|   Note: |  |  |  |  |  |  |  |  |  |  |  |  |  |  |  |  |  |
| --- | --- | --- | --- | --- | --- | --- | --- | --- | --- | --- | --- | --- | --- | --- | --- | --- | --- | --- | --- | --- | --- | --- | --- | --- | --- | --- | --- | --- | --- | --- | --- | --- | --- | --- | --- | --- | --- | --- | --- | --- | --- | --- | --- | --- | --- | --- | --- | --- | --- | --- | --- | --- | --- | --- | --- | --- | --- | --- | --- | --- | --- | --- | --- | --- | --- | --- | --- | --- | --- | --- | --- | --- | --- | --- | --- | --- | --- | --- | --- | --- | --- | --- | --- | --- | --- | --- | --- | --- | --- | --- | --- | --- | --- | --- | --- | --- | --- | --- | --- | --- | --- | --- | --- | --- | --- | --- | --- | --- | --- | --- | --- | --- | --- | --- | --- | --- | --- | --- | --- | --- | --- | --- | --- | --- | --- | --- | --- | --- | --- | --- | --- | --- | --- | --- | --- | --- | --- | --- | --- | --- | --- | --- | --- | --- | --- | --- | --- | --- | --- | --- | --- | --- | --- | --- | --- | --- | --- | --- | --- | --- | --- | --- | --- | --- | --- | --- | --- | --- | --- | --- | --- | --- | --- | --- | --- | --- | --- | --- | --- | --- | --- | --- | --- | --- | --- | --- | --- | --- | --- | --- | --- | --- | --- | --- | --- | --- | --- | --- | --- | --- | --- | --- | --- | --- | --- | --- | --- | --- | --- | --- | --- | --- | --- | --- | --- | --- | --- | --- | --- | --- | --- | --- | --- | --- | --- | --- | --- | --- | --- | --- | --- | --- | --- | --- | --- | --- | --- | --- | --- | --- | --- | --- | --- | --- | --- | --- | --- | --- | --- | --- | --- | --- | --- | --- | --- | --- | --- | --- | --- | --- | --- | --- | --- | --- | --- | --- | --- | --- | --- | --- | --- | --- | --- | --- | --- | --- | --- | --- | --- | --- | --- | --- | --- | --- | --- | --- | --- | --- | --- | --- | --- | --- | --- | --- | --- | --- | --- | --- | --- | --- | --- | --- | --- | --- | --- | --- | --- | --- | --- | --- | --- | --- | --- | --- | --- | --- | --- | --- | --- | --- | --- | --- | --- | --- | --- | --- | --- | --- | --- | --- | --- | --- | --- | --- | --- | --- | --- | --- | --- | --- | --- | --- | --- | --- | --- | --- | --- | --- | --- | --- | --- | --- | --- | --- | --- | --- | --- | --- | --- | --- | --- | --- | --- | --- | --- | --- | --- | --- | --- | --- | --- | --- | --- | --- | --- | --- | --- | --- | --- | --- | --- | --- | --- | --- | --- | --- | --- | --- | --- | --- | --- | --- | --- | --- | --- | --- | --- | --- | --- | --- | --- | --- | --- | --- | --- | --- | --- | --- | --- | --- | --- | --- | --- | --- | --- | --- | --- | --- | --- | --- | --- | --- | --- | --- | --- | --- | --- | --- | --- | --- | --- | --- | --- | --- | --- | --- | --- | --- | --- | --- | --- | --- | --- | --- | --- | --- | --- | --- | --- | --- | --- | --- | --- | --- | --- | --- | --- | --- | --- | --- | --- | --- | --- | --- | --- | --- | --- | --- | --- | --- | --- | --- | --- | --- | --- | --- | --- | --- | --- | --- | --- | --- | --- | --- | --- | --- | --- | --- | --- | --- | --- | --- | --- | --- | --- | --- | --- | --- | --- | --- | --- | --- | --- | --- | --- | --- | --- | --- | --- | --- | --- | --- | --- | --- | --- | --- | --- | --- | --- | --- | --- | --- | --- | --- | --- | --- | --- | --- | --- | --- | --- | --- | --- | --- | --- | --- | --- | --- | --- | --- | --- | --- | --- | --- | --- | --- | --- | --- | --- | --- | --- | --- | --- | --- | --- | --- | --- | --- | --- | --- | --- | --- | --- | --- | --- | --- | --- | --- | --- | --- | --- | --- | --- | --- | --- | --- | --- | --- | --- | --- | --- | --- | --- | --- | --- | --- | --- | --- | --- | --- | --- | --- | --- | --- | --- | --- | --- | --- | --- | --- | --- | --- | --- | --- | --- | --- | --- | --- | --- | --- | --- | --- | --- | --- | --- | --- | --- | --- | --- | --- | --- | --- | --- | --- | --- | --- | --- | --- | --- | --- | --- | --- | --- | --- | --- | --- | --- | --- | --- | --- | --- | --- | --- | --- | --- | --- | --- | --- | --- | --- | --- | --- | --- | --- | --- | --- | --- | --- | --- | --- | --- | --- | --- | --- | --- | --- | --- | --- | --- | --- | --- | --- | --- | --- | --- | --- | --- | --- | --- | --- | --- | --- | --- | --- | --- | --- | --- | --- | --- | --- | --- | --- | --- | --- | --- | --- | --- | --- | --- | --- | --- | --- | --- | --- | --- | --- | --- | --- | --- | --- | --- | --- | --- | --- | --- | --- | --- | --- | --- | --- | --- | --- | --- | --- | --- | --- | --- | --- | --- | --- | --- | --- | --- | --- | --- | --- | --- | --- | --- | --- | --- | --- | --- | --- | --- | --- | --- | --- | --- | --- | --- | --- | --- | --- | --- | --- | --- | --- | --- | --- | --- | --- | --- | --- | --- | --- | --- | --- | --- | --- | --- | --- | --- | --- | --- | --- | --- | --- | --- | --- | --- | --- | --- | --- | --- | --- | --- | --- | --- | --- | --- | --- | --- | --- | --- | --- | --- | --- | --- | --- | --- | --- | --- | --- | --- | --- | --- | --- | --- | --- | --- | --- | --- | --- | --- | --- | --- | --- | --- | --- | --- | --- | --- | --- | --- | --- | --- | --- | --- | --- | --- | --- | --- | --- | --- | --- | --- | --- | --- | --- | --- | --- | --- | --- | --- | --- | --- | --- | --- | --- | --- | --- | --- | --- | --- | --- | --- | --- | --- | --- | --- | --- | --- | --- | --- | --- | --- | --- | --- | --- | --- | --- | --- | --- | --- | --- | --- | --- | --- | --- | --- | --- | --- | --- | --- | --- | --- | --- | --- | --- | --- | --- | --- | --- | --- | --- | --- | --- | --- | --- | --- | --- | --- | --- | --- | --- | --- | --- | --- | --- | --- | --- | --- | --- | --- | --- | --- | --- | --- | --- | --- | --- | --- | --- | --- | --- | --- | --- | --- |

Note: OR – odd ratio; CI – confidence interval; p-value equal to “0.000” means p-value<0.001.

^a^ Cluster 1: Austria, Cyprus, Greece, Estonia, Germany, and Spain. Cluster 2: Finland and Luxembourg. Cluster 3:Belgium, Bulgaria, Latvia, Malta, and Slovakia. Cluster 4: Denmark, Hungary, Lithuania, and Sweden. Clusters 5&6: Croatia, Italy, Slovenia, Romania, and Portugal. Cluster 7: Czechia, Ireland, Netherlands, and Poland. ^b^ percentage of people reporting the use of OTC drugs. ^c^ rx – prescribed. ^d^ unmet means unmet need for health care followed by the motive. ^e^ bmi - body mass index. ^f^ Country codes: AT Austria, BE Belgium, BG Bulgaria, CY Cyprus, CZ Czechia, DE Germany, DK Denmark, EE Estonia, EL Greece, ES Spain, FI Finland, HR Croatia, HU Hungary, IE Ireland, IT Italy, LT Lituania, LU Luxembourg, LV Latvia, MT Malta, NL Netherlands, PL Poland, PT Portugal, RO Romania, SE Sweden, SI Slovenia, SK Slovakia
